# Supplementary material for: Water-generated dangling linkers in a metal-organic framework
Source: Nat Commun. 2026 Mar 11;17:3805. doi: 10.1038/s41467-026-70247-z (PMC13111590; doi:10.1038/s41467-026-70247-z)
Supplement: Supplementary file 1 — Supplementary Information [file 41467_2026_70247_MOESM1_ESM.pdf]

Supporting Information for

Water-generated dangling linkers in a metal-organic framework

Yao Fu[1,2], Yifeng Yao[3], Subhradip Paul[1], Kenji Mochizuki[3*], Gaël De Paëpe[1*]

[1] Univ. Grenoble Alpes, CEA, IRIG-MEM, 38000 Grenoble, France

[2] Department of Chemistry, Fudan University, 200438 Shanghai, PR China

[3] Department of Chemistry, Zhejiang University, 310027 Hangzhou, PR China

E-mail: kenji_mochizuki@zju.edu.cn; gael.depaepe@cea.fr

Supplementary Table S1. Measured ^{1}H spin-lattice relaxation times (T_1) for all samples using the inversion-recovery pulse sequence.

T_1_^{1}H (s)	Fresh	26 H_2O/u.c.	160 H_2O/u.c.	320 H_2O/u.c.	Dried
Aromatic H	0.19	0.19	0.48	0.54	0.19
H_2O	/	0.21	0.42	0.44	/
μ_3-OH	0.20	0.20	0.39	0.39	0.2

Supplementary Table S2. Experimental ^{13}C chemical shifts of the "320 H_2O/u.c." sample, together with the calculated ^{13}C chemical shifts for both the intact and dangling linker structures, based on the configurations shown in Figure 3 and Figure S8, S9. All carbon atoms are labeled in each configuration, and the corresponding ^{13}C chemical shift values are summarized in this table.

Experimental data for sample "320 H_2O/u.c."	
peak	^{13}C chemical shift (ppm)
C1	171.5
C2	137.1
C3	128.2
C4	175.1

Calculated data for intact linker	
position	^{13}C chemical shift (ppm)
C1	173.4
C2	133.2
C3	127.9

Calculated ^{13}C chemical shift (ppm) for Case A						
position	A-I (COO- + $2H_2O$)	A-II (COOH + OH- + void-1)	A-II (COOH + OH- + void-2)	A-III (COO- + H_2O)	A-IV (COOH + OH- + H_2O-1)	A-IV (COOH + OH- + H_2O-2)
a	175.9	176.6	176.4	176.2	176.1	176.3
b	132.1	135.9	136.5	131.0	136.1	136.2
c	128.6	130.0	130.0	128.9	129.9	129.8
d	128.4	128.6	128.6	128.1	128.6	128.6
e	135.6	130.6	129.8	135.4	130.6	130.3
f	128.0	129.3	129.7	126.9	129.6	129.6
g	128.1	128.0	128.2	127.9	128.3	128.2
h	180.8	173.4	174.0	176.5	174.1	174.1

Calculated ^{13}C chemical shift (ppm) for Case B					
position	B-I (COO- + $2H_2O$)	B-II (COOH + OH- + void)	B-III (COO- + H_2O)	B-IV (COOH + OH- + H_2O)	B-V (COOH + OH-)
a	175.0	177.5	177.0	176.7	191.5
b	130.3	135.4	135.3	132.1	141.5
c	128.1	126.9	127.4	129.2	140.3
d	127.0	127.0	126.0	128.4	137.4
e	134.3	129.3	133.4	131.9	150.5
f	128.6	129.5	132.1	131.2	139.2
g	128.7	128.0	128.9	129.7	140.5
h	178.9	176.3	176.8	174.5	187.7

Supplementary Table S3. Calculated ^{1}H chemical shifts of all μ3-OH protons based on the configurations shown in Figures 3, S11, and S12. The μ3-OH protons are labeled as illustrated in the two examples shown below.

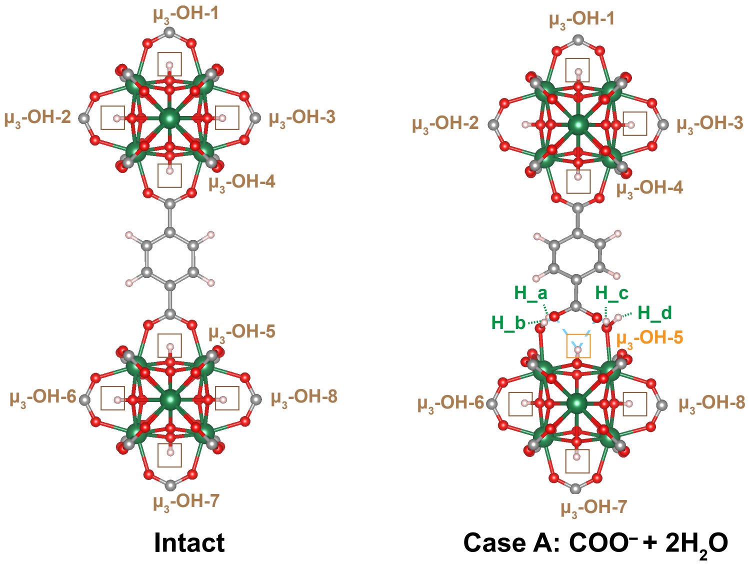

Calculated μ3-OH ^{1}H chemical shift (ppm) for Case A						
μ3-OH position	A-I (COO- + 2H2O)	A-II (COOH + OH- + void-1)	A-II (COOH + OH- + void-2)	A-III (COO- + H2O)	A-IV (COOH + OH- + H2O-1)	A-IV (COOH + OH- + H2O-2)
1	1.76	1.82	1.81	1.78	1.82	1.82
2	1.75	1.79	1.80	1.75	1.77	1.80
3	1.22	1.32	1.30	1.25	1.31	1.31
4	1.81	1.80	1.82	1.82	1.84	1.82
5	5.94	3.59	3.07	6.70	2.69	2.57
6	2.20	2.19	2.19	2.11	1.90	1.86
7	1.87	1.73	1.78	1.80	1.83	1.78
8	2.18	2.11	1.78	2.16	1.91	1.82

Calculated μ3-OH ^{1}H chemical shift (ppm) for Intact and Case B						
μ3-OH position	Intact	B-I (COO- + 2H2O)	B-II (COOH + OH- + void)	B-III (COO- + H2O)	B-IV (COOH + OH- + H2O)	B-V (COOH + OH-)
1	2.07	1.98	1.95	1.87	1.91	1.82
2	2.08	2.28	2.23	1.83	2.25	1.84
3	2.12	2.39	2.25	2.00	2.74	2.15
4	2.08	2.18	2.26	2.04	2.13	2.03
5	2.12	1.81	2.53	1.96	1.97	1.89
6	2.08	2.82	1.95	2.38	2.39	2.45
7	2.07	2.00	1.81	1.93	1.84	1.75
8	2.08	2.81	2.61	2.49	1.99	2.31

Supplementary Table S4. Calculated ^{1}H chemical shifts for water-related protons. In the COO- + $2H_2O$ configuration of Case A (Table S3), water-related protons are specifically labeled, while the remaining water-related protons are labeled across all configurations in Figures S11 and S12.

Calculated ^{1}H chemical shifts of water-related protons (in green) for Case A						
position	A-I (COO- + $2H_2O$)	A-II (COOH + OH- + void-1)	A-II (COOH + OH- + void-2)	A-III (COO- + H_2O)	A-IV (COOH + OH- + H_2O-1)	A-IV (COOH + OH- + H_2O-2)
H_a	3.61	8.09	8.148	3.76	8.18	8.16
H_b	10.72	4.81	5.57	14.766	3.36	2.72
H_c	10.12	/	/	/	13.60	15.22
H_d	3.52	/	/	/	3.80	3.91

Calculated ^{1}H chemical shifts of water-related protons (in green) for Case B					
position	B-I (COO- + $2H_2O$)	B-II (COOH + OH- + void)	B-III (COO- + H_2O)	B-IV (COOH + OH- + H_2O)	B-V (COOH + OH-)
H_a	3.66	5.66	9.63	5.82	15.11
H_b	13.41	11.56	3.47	11.09	4.86
H_c	12.53	/	/	11.18	/
H_d	3.71	/	/	3.53	/

Supplementary Figures

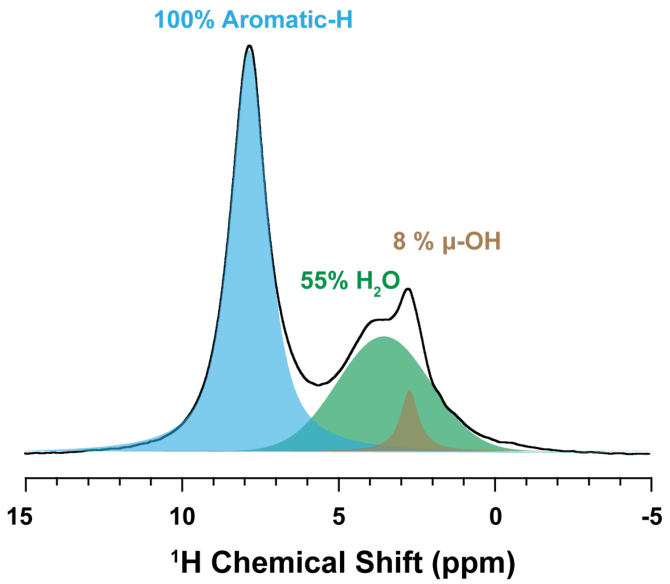

Figure S1. To calculate the adsorbed water content in MOF one unit cell, consider the following example: If the aromatic H is 100%, then for a unit cell $Zr_{24}O_{16}(OH)_{16}(BDC)_{24}$, it would be 100%/4/24 = 1.04%, as each BDC^{2-} linker has 4 aromatic protons. For H_2O protons is 55%, each H_2O molecule accounts for 55%/2 = 27.5%. Therefore, the sample would be named as 27.5/1.04 = 26 H_2O/u.c.

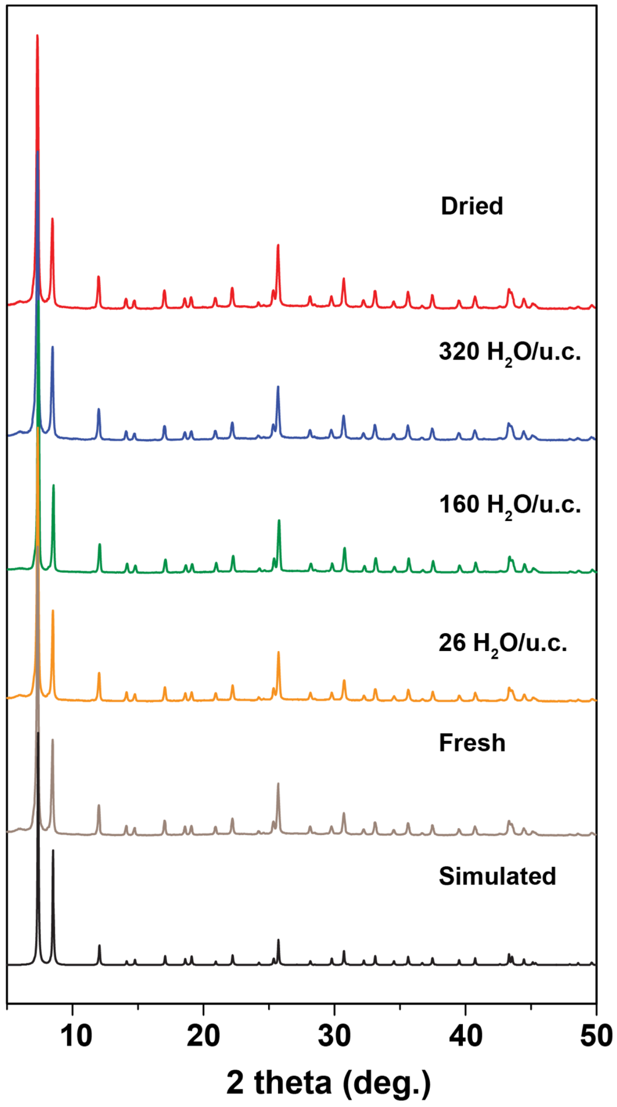

Figure S2. X-ray powder diffraction patterns of simulated UiO-66, UiO-66 samples with different water contents in the pores.

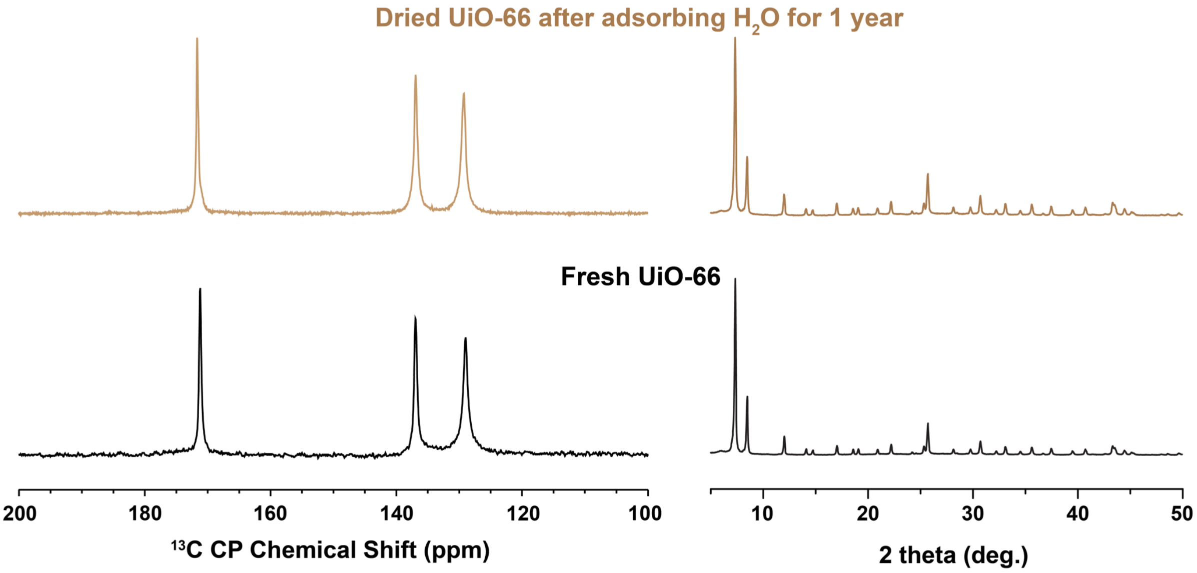

Figure S3. (left) ^{13}C cross-polarization spectra, and (right) X-ray powder diffraction patterns on fresh UiO-66 (black) and dried UiO-66 after adsorbing H_2O for 1 year (yellow). The near-identical XRD patterns and ^{13}C NMR spectra of these two samples demonstrate the resilience and structural stability of UiO-66 under prolonged aqueous conditions.

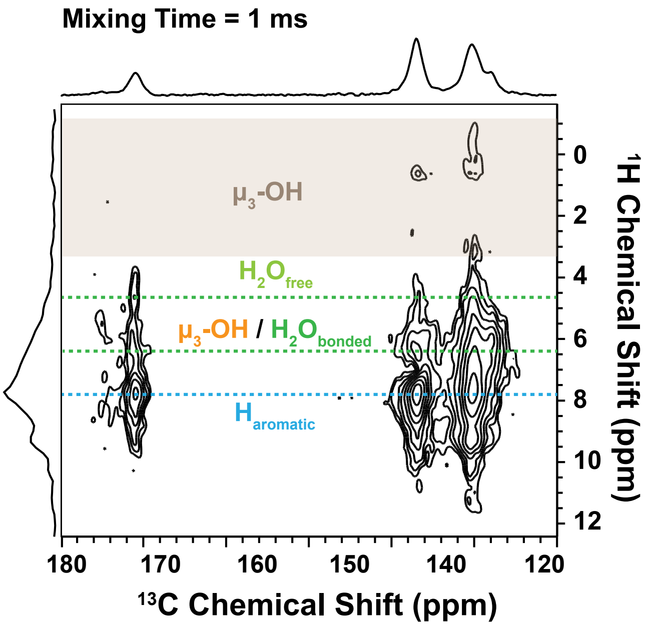

Figure S4. ^{1}H-^{13}C HETCOR NMR spectrum recorded at room temperature on the "160 H_2O/u.c." sample with a mixing time of 1 ms. The results are similar to those obtained with a 5 ms mixing time, as shown in Figure 2b.

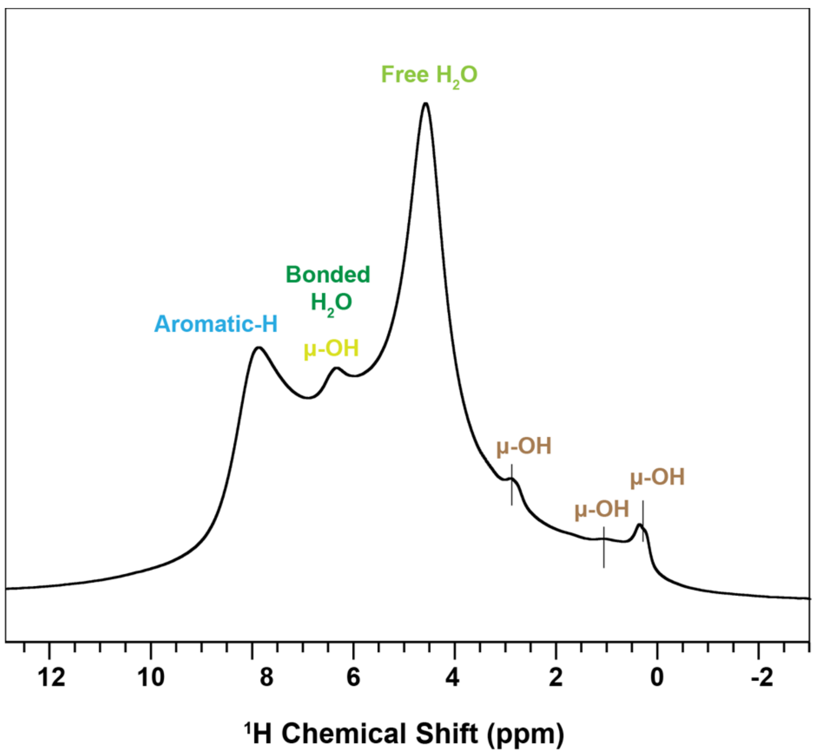

Figure S5. The ^1H one-pulse spectrum of the 160 H_2O/u.c. sample, recorded at a spinning rate of 35 kHz, provides improved ^1H resolution compared to the spectrum shown in Figure 1b (recorded at a spinning rate of 10 kHz). This enhanced resolution reveals two distinct H_2O environments: free H_2O at 4.7 ppm and hydrogen-bonded water at 6.4 ppm. Additionally, three distinct μ_3-OH sites are identified within the 0-3 ppm range.

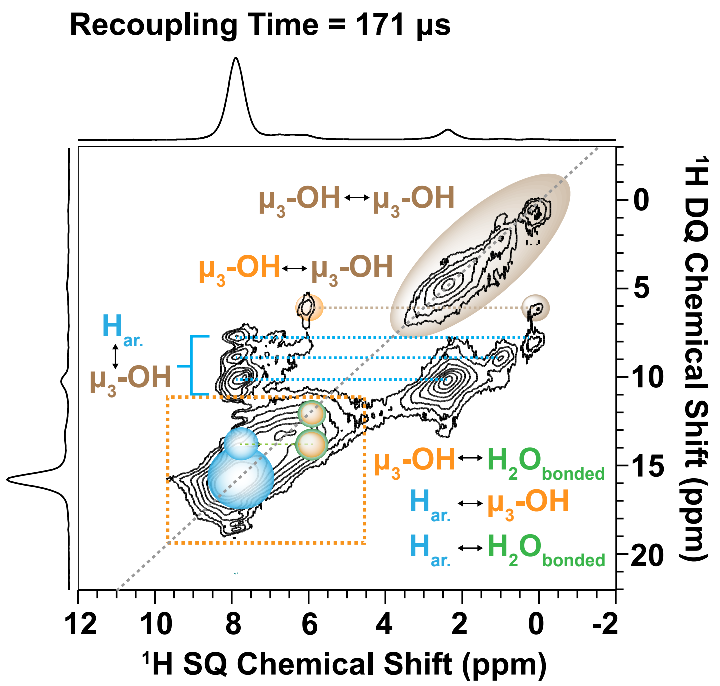

Figure S6. ^{1}H-^{1}H 2D double-quantum single-quantum (DQ-SQ) correlation spectrum acquired at room temperature on the "160 H_2O/u.c." sample with a recoupling time of 171 μs. The results are similar to those obtained with a 57 μs recoupling time, as shown in Figure 2c. The dotted orange region shows spatial correlations arising from the proximity of aromatic protons, the μ_3-OH group near the detached linker, and hydrogen-bonded water, along with their respective self-correlations.

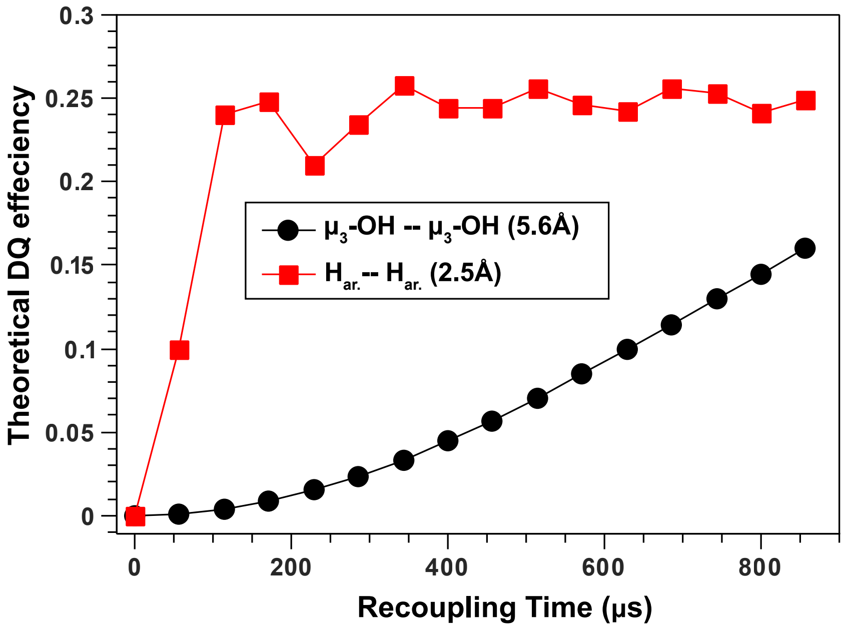

Figure S7. SIMPSON simulations of ^{1}H-^{1}H 2D double-quantum (DQ) recoupling efficiency using the BABA sequence at varying dipolar distances. For a recoupling time of 57 μs, transfer between aromatic protons separated by ~2.5 Å is predicted to be ~100 times stronger than between the closest μ_3-OH protons (~5.6 Å apart). For 171 μs, transfer between aromatic protons (~2.5 Å) is predicted to be ~30 times stronger than between the closest μ_3-OH protons (~5.6 Å apart).

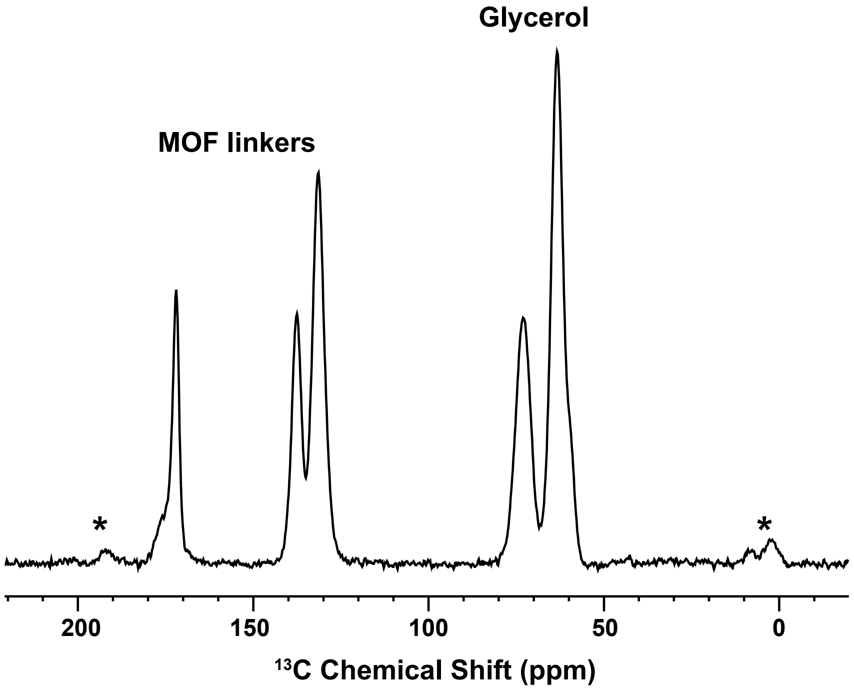

Figure S8. 1D {^{1}H-}^{13}C CPMAS spectrum of the UiO-66 DNP sample prepared with the polarizing agent AMUPol solution (consisting of 60% d_6-glycerol, 30% D_2O, and 10% H_2O by volume). Signals from ^{1}H/^{13}C nuclei in AMUPol are generally not detectable because the nuclei are too close to unpaired electrons, leading to significant broadening of the resonances due to hyperfine coupling. However, the ^{13}C signals from glycerol, appearing in the 50-80 ppm range, remain observable. The asterisk (*) indicates the spinning sideband signal.

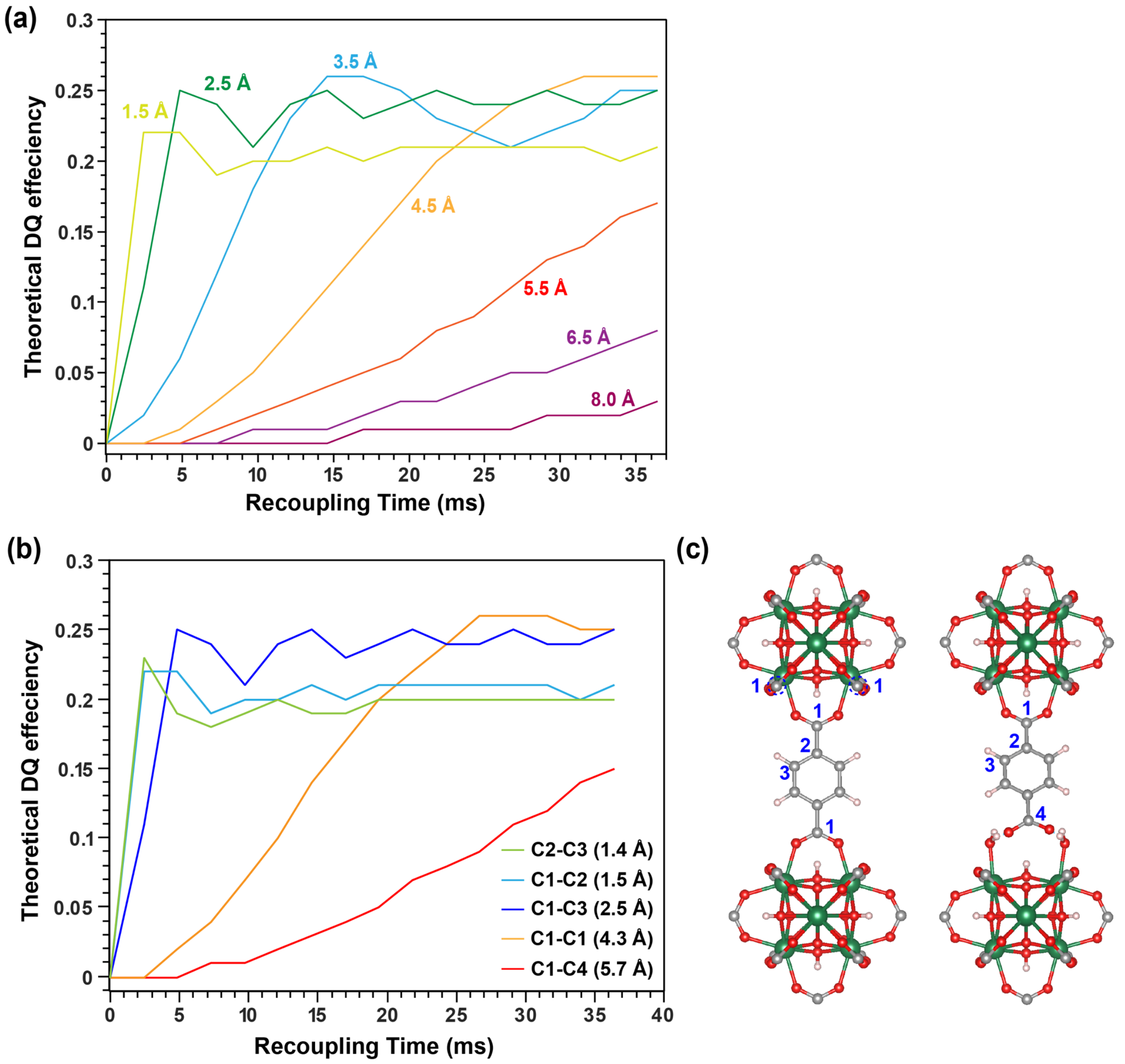

Figure S9. (a) SIMPSON simulations showing the ^{13}C-^{13}C 2D double quantum (DQ) recoupling efficiency curve for the S3 recoupling sequence under varying ^{13}C-^{13}C dipolar distances. (b) SIMPSON simulations of the ^{13}C-^{13}C 2D DQ recoupling efficiency curve for the S3 sequence at specific ^{13}C-^{13}C distances, as indicated in (c). Note that the C1-C1 distance refers to closest neighboring linkers, rather than the opposite carbons within the same linker. The numerical simulations were done with 2 spins. The simulations shown in (a) was done with chemical shifts of 5 ppm and -5 ppm of isotropic chemical shifts. The simulations in (b) were done with experimental chemical shifts of the respective carbons. For every simulation 233 pairs of α and β Euler angles were used based on Zaremba, Conroy and Wolfsburg (ZCW) scheme. For each of 233 pairs, 48 γ angles were used for the carousel averaging[1–4].

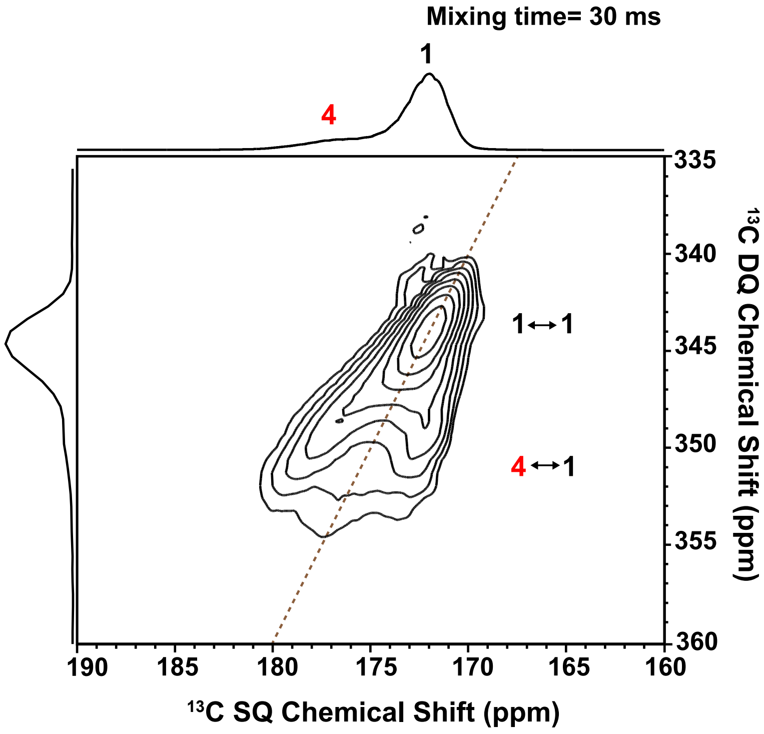

Figure S10. DNP-enhanced ^{13}C-^{13}C 2D double-quantum single-quantum (DQ-SQ) correlation spectra of UiO-66 sample recorded at 100 K with mixing time of 30 ms.

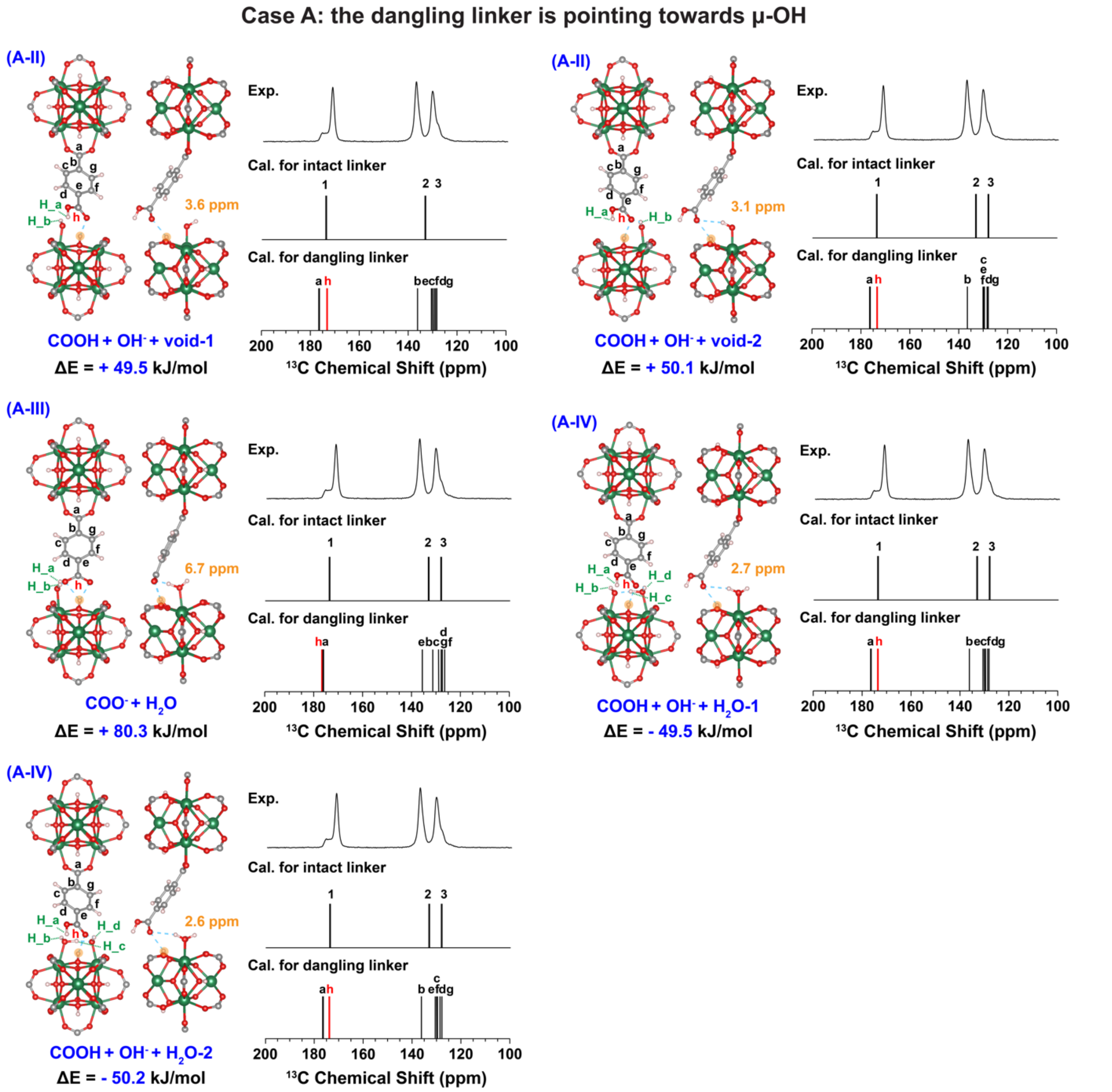

Figure S11. (Left) Various dangling linker configuration structures based on case A (where the dangling linker points toward μ_3-OH) are presented, including front and side views optimized through DFT calculations. Green, red, grey, and white spheres represent Zr, O, C, and H atoms, respectively, with hydrogen bonding depicted by blue dashed lines. The quantum mechanical calculations provide the energies for each configuration. (Right) Experimental ^{13}C CPMAS spectrum of the "320 H_2O/u.c." sample and the calculated ^{13}C chemical shifts of intact linker (structure shown in Figure 3a) and dangling linker structure based on configurations on the left. The calculated ^{1}H chemical shifts for the labeled μ_3-OH groups (highlighted in orange) in all the configurations are shown. The calculated ^{1}H chemical shifts of the water- related protons highlighted in green for all configurations are provided in Table S4.

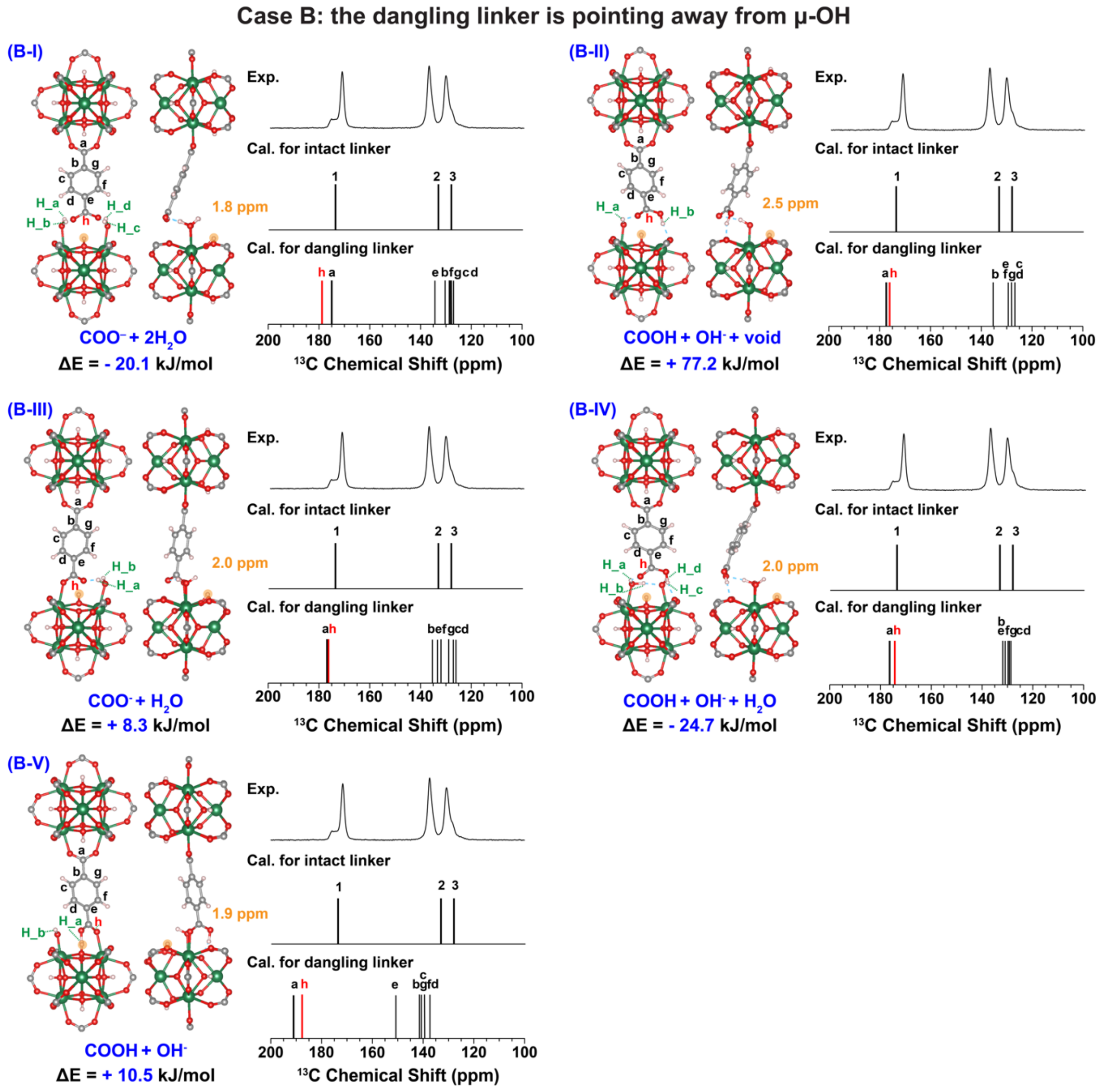

Figure S12. (Left) Various dangling linker configuration structures based on case B (where the dangling linker points away from μ_3-OH) are presented, including front and side views optimized through DFT calculations. Green, red, grey, and white spheres represent Zr, O, C, and H atoms, respectively, with hydrogen bonding depicted by blue dashed lines. (Right) Experimental ^{13}C CPMAS spectrum of the "320 H_2O/u.c." sample and the calculated ^{13}C chemical shifts of intact linker (structure shown in Figure 3a) and dangling linker structure based on configurations on the left. The calculated ^{1}H chemical shifts for the labeled μ_3-OH groups (highlighted in orange) in all the configurations are shown. The calculated ^{1}H chemical shifts of the water- related protons highlighted in green for all configurations are provided in Table S4.

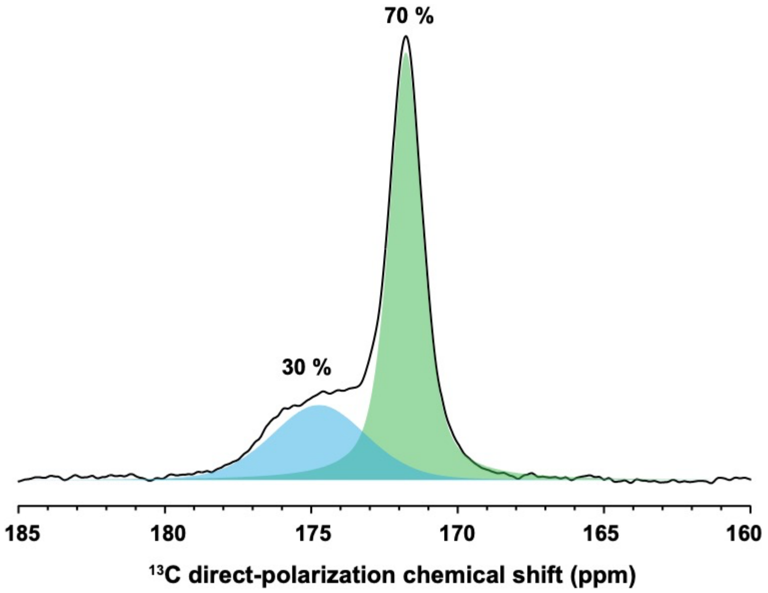

Figure S13. ^{13}C direct-polarization (DP) spectrum obtained of the “160 H_2O/u.c.” UiO-66 sample. The UiO-66 MOF was synthesized using ^{13}C-labeled carboxylate groups on the H_4BDC linker. For the DP experiments on the “160 H_2O/u.c.” sample, a recycle delay of 200 s was used, based on a measured ^{13}C T_1 of 38 s at room temperature. The resulting spectrum accurately reflects the intensity ratio of the two carboxylate groups: the left peak (C4) corresponds to dangling-linker carboxylates, and the right peak (C1) corresponds to the original carboxylate groups.

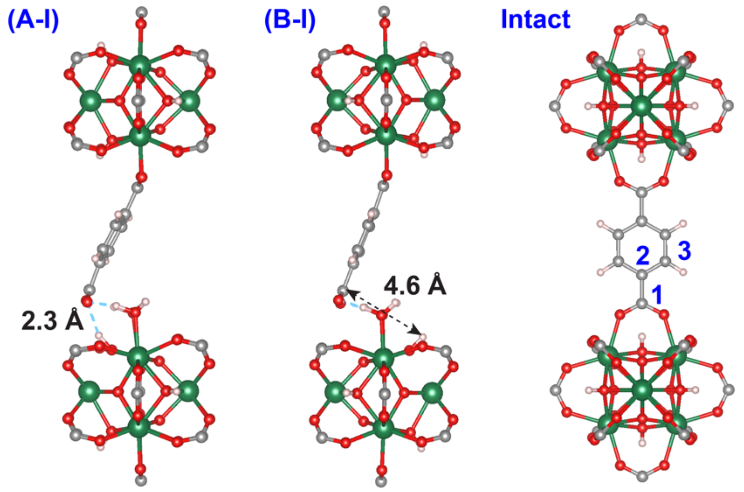

Figure S14. The distance between the dangling carboxylate carbon and the nearest μ_3-OH proton is approximately 2.3 Å in configuration (A-I) and 4.6 Å in configuration (B-I) for the COO^{-}+$2H_2O$ configuration system. For the intact linker, the distances between μ_3-OH and the nearest carbon atoms C1, C2, and C3 of the intact linker are approximately 2.6 Å, 3.8 Å, and 4.5 Å, respectively.

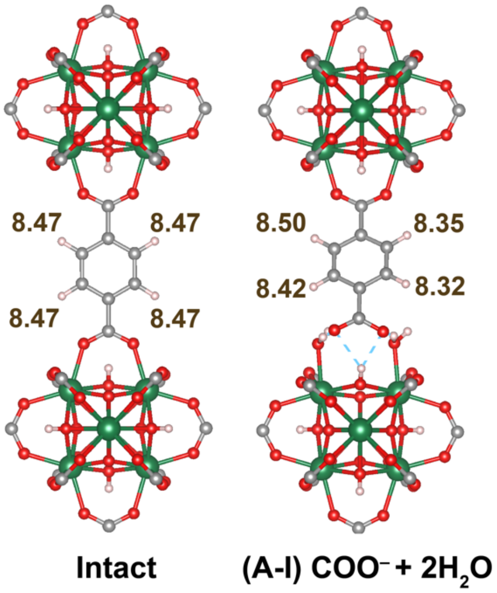

Figure S15. The calculated ^{1}H chemical shifts of intact linker and dangling linker structure based on configurations shown in (A-I).

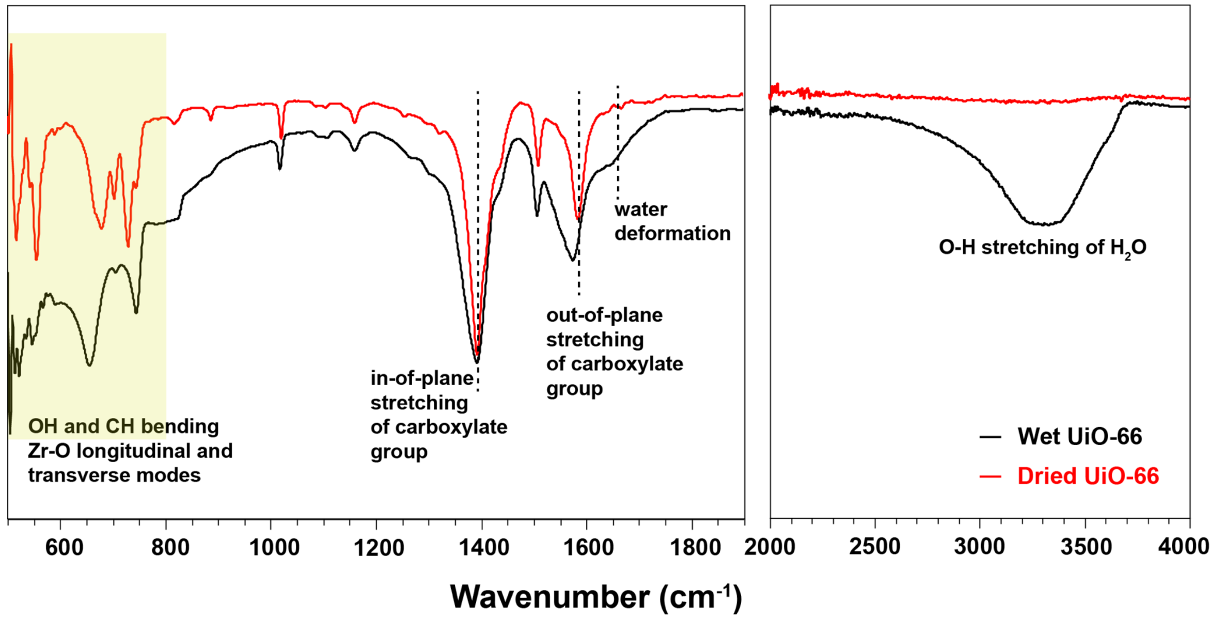

Figure S16. FT-IR spectra of the dried UiO-66 sample (red curve) and the wet UiO-66 sample (black curve). Peaks in the range of 400-800 cm^{-1} are attributed to OH and CH bending as well as Zr-O longitudinal and transverse vibrational modes. The peaks at 1395 cm^{-1} and 1589 cm^{-1} correspond to the in-plane and out-of-plane stretching vibrations of the carboxylate group, respectively. The peak at 1660 cm^{-1} is assigned to water deformation, while the peak at 3300 cm^{-1} is associated with OH stretching of H_2O.

References

(1) Bak, M.; Rasmussen, J. T.; Nielsen, N. C. SIMPSON: A General Simulation Program for Solid-State NMR Spectroscopy. *J. Magn. Reson.* **2000**, *147* (2), 296–330.

(2) Zaremba, S. K. Good Lattice Points, Discrepancy, and Numerical Integration. *Ann. Mat. Pura Ed Appl.* **1966**, *73* (1), 293–317.

(3) Conroy, H. Molecular Schrödinger Equation. VIII. A New Method for the Evaluation of Multidimensional Integrals. *J. Chem. Phys.* **1967**, *47* (12), 5307–5318.

(4) Cheng, V. B.; Suzukawa, H. H.; Wolfsberg, M. Investigations of a Nonrandom Numerical Method for Multidimensional Integration. *J. Chem. Phys.* **1973**, *59* (8), 3992–3999.
